# A novel screening instrument for the assessment of autism in German language: validation of the German version of the RAADS-R, the RADS-R

**DOI:** 10.1007/s00406-024-01894-w

**Published:** 2024-10-27

**Authors:** Jördis Rausch, Thomas Fangmeier, Christine M. Falter-Wagner, Helene Ackermann, Julia Espelöer, Lars P. Hölzel, Andreas Riedel, Ariella Ritvo, Kai Vogeley, Ludger Tebartz van Elst

**Affiliations:** 1 Department of Psychiatry and Psychotherapy, Medical Center, Faculty of Medicine, University of Freiburg, University of Freiburg, Freiburg, Germany; 2 Department of Psychiatry and Psychotherapy, Faculty of Medicine and University Hospital Cologne, University of Cologne, Cologne, Germany; 3 Department of Psychiatry and Psychotherapy, Medical Faculty, LMU Munich, Munich, Germany; 4 Clinical Faculty, Yale University School Of Medicine, New Haven, USA; 5 Health Services Research, Oberberg Gruppe, Berlin, Germany; 6 Luzerner Psychiatrie, Ambulante Dienste, Lucerne, Switzerland; 7 Department of Psychiatry and Psychotherapy, University Medical Center of the Johannes Gutenberg-University Mainz, Mainz, Germany

**Keywords:** Autism, ASD, Neurodevelopmental disorders, Diagnostic, Adults, Questionnaire

## Abstract

The Ritvo Autism Asperger Diagnostic Scale-Revised (RAADS-R) demonstrated excellent results in its original study, with a sensitivity of 97% and a specificity of 100% (Ritvo et al. in J Autism Dev Disord 41:1076–1089, 2011). As a result, it was included in the National Institute for Health and Care Excellence (NICE) guidelines (Recommendations | Autism spectrum disorder in adults: diagnosis and management | Guidance | NICE, 2022). The questionnaire includes 80 questions across four subcategories (language, social relatedness, circumscribed interests, sensory motor). So far, the subcategory sensory motor has not been addressed in most available instruments, despite being part of the diagnostic criteria specified in DSM-5 (Falkai et al., in Diagnostisches Und Statistisches Manual Psychischer Störungen DSM-5. Hogrefe, 2015) and ICD-11 (ICD-11 for Mortality and Morbidity Statistics, 2022). In our validation study, we tested a translated German version of the questionnaire in 299 individuals (110 persons with ASD according to ICD-10 F84.0, F84.5, 64 persons with an primary mental disorders (PMD), 125 persons with no disorders). To enhance the practical use of the instrument in clinical everyday practice, the questionnaire was completed by the participants without the presence of a clinician—unlike the original study. Psychiatric diagnoses were established following the highest standards, and psychometric properties were calculated using established protocols. The German version of the RADS-R yielded very good results, with a high sensitivity of 92.5% and a high specificity of 93.6%. The area under the curve (*AUC* = 0.976), indicates a high quality and discriminatory power of RADS-R. Furthermore, the ROC curve analysis showed that the optimal threshold to distinguish between the ASD and non-ASD groups in the German version of the RAADS-R is a score of 81. In comparison to the RADS-R, the co-administered instruments Social Responsiveness Scale (SRS), Autism Spectrum Quotient (AQ), and Empathy Quotient (EQ) each showed slightly better specificity but worse sensitivity in this sample.The study included individuals already diagnosed with ASD according to ICD-10 (F84.0, F84.5), with or without an primary mental disorders, preventing us from identifying the influence of comorbidities on the RADS-R results. In addition, a self-report questionnaire has generally only limited objectivity and may allow for false representation of the symptoms. The RADS-R compares well with other questionnaires and can provide valuable additional information. It could turn out to be a helpful diagnostic tool for patients in Germany. We propose naming the German version RADS-R (Ritvo Autism Diagnostic Scale – rRevised) to reflect the change in terminology.

## Introduction

During the past years we can observe an increase of the diagnosis of autism spectrum disorder (ASD). While the prevalence was estimated at four in 10,000 people in 1989 [5], more recent studies in 2011 estimate a prevalence of one in 100 people [6, 7]. Whether this is due to an actual increase in the disorder, due to subtle changes in the cut-off criteria, or due to a shift in the focus of mental health professionals has been the subject of many debates. However, it is clear that the skillset required to diagnose a patient with ASD is not sufficiently trained in relevant medical professions. A survey carried out among service users in the United Kingdom highlighted the lack of knowledge by general practitioners as one of the barriers to diagnosis [8]. Yet, a diagnosis is essential to obtain social support as well as treatment within the healthcare system. A formal diagnosis allows people with ASD to apply for the formal recognition of disability, adaptations of their working conditions, and specialized training. Specialized treatment for adults with ASD is not widely available, but programs such as the FASTER (“Freiburger Asperger-Spezifische Therapie für ERwachsene” = Freiburg Asperger-specific therapy for adults) and EVA&SCOTT (“Social Cognition Training Tool”, and its enhancement “Emotionen Verstehen und Ausdruecken” = understanding and expressing emotions) have been developed and validated in phase I/II studies and are currently under examination in a multicenter, controlled phase III trial [9–13]. Other programs, such as the GATE manual, have also been created for group settings [14]. Data from the Expectations to Psychotherapy Autism Spectrum (EPAS) questionnaire, which was developed to assess the requirements and needs of persons with ASD with regards to psychotherapy, highlighted the need to tailor psychotherapeutic concepts to autism-related deficits [15]. Autistic traits, already a diagnostic challenge themselves, can lead to the development of comorbid axis I symptoms such as social anxiety [16]. Therefore, people with ASD present with a higher rate of co-occurring mental health conditions such as ADHD (28%), anxiety disorders (20%), depressive disorders (9%), and schizophrenia (4%) compared to the general population [17]. This further complicates ASD diagnosis, as comorbidities do not automatically rule out the diagnosis of ASD, while overlapping symptoms can make the evaluation of ASD-related symptoms more difficult.

Efforts to identify objective biological markers have shown s an autism-associated increase in the albumin quotient in the cerebrospinal fluid [18], but other biological parameters, such as rate of intermittent rhythmic delta or theta activity (IRDA/IRTA) before HV in an EEG, have not shown difference between autistic and non-autistic participants [19]. Currently, no biological markers have sufficient sensitivity and specificity to be used as diagnostic tools in everyday practice. Thus, a clear-cut biological indicator with sufficient universality (i.e., applicable to the whole ASD population and not just a sub-group of for example a certain genetic disorder) has yet to be identified. Therefore, the current diagnostic procedure has to rely solely on clinical interviews by trained professionals. To standardize clinical interviews, the Developmental, Dimensional and Diagnostic Interview—Adult Version (3Di-Adult) was developed [20]. For testing individuals without a learning disability, the UK guidelines of the National Institute for Health and Care Excellence (NICE) recommends to apply the Ritvo Autism Asperger Diagnostic Scale—Revised (RAADS-R) as a screening instrument and the Adult Asperger Assessment (AAA) as a structured interview which is an interview essentially based on the Autism Spectrum Quotient (AQ) and Empathy Quotient (EQ) [1, 2, 21]. In terms of standardized tests NICE recommends the Autism Diagnostic Observation Schedule—Generic (ADOS-G) as well as the Asperger Syndrome (and High-Functioning Autism) Diagnostic Interview (ASDI) [2, 22, 23]. Apart from the RAADS all tools are available in the German language. Locally other questionnaires such as the Freiburg Questionnaire of linguistic pragmatics (FQLP) or the Bermond–Vorst Alexithymia Questionnaire (BVAQ) may also be useful as additional tools [24, 25]. It has to be noted that even carefully developed instruments such as the ADOS-G upon further inspection do have a higher proportion of false negative diagnosis in females than in males [26]. Recognizing that females might experience and express their symptoms differently the Girls Questionnaire for Autism Spectrum Condition (GQ-ASC) was modified for adults [27]. To adjust for compensation effects in the sense of camouflaging, a trait more common in females with ASD, the Camouflaging Autistic Traits Questionnaire (CAT-Q) was developed [28]. Since ASD is a neurodevelopmental disorder and its characteristics can overlap with other mental disorders, such as schizophrenia and depression, verifying the onset of symptoms in childhood is crucial. For adults, who were not assessed in their childhood or adolescence, this is often achieved by obtaining a thorough developmental history (e.g. Autism Diagnostic Interview Revised (ADI-R) [29]). Psychometric instruments such as the “Australian Scale for Asperger’s Syndrome (ASAS)” [30] may provide additional information. Further childhood documents like school reports or photographs may be helpful [2]. The currently very high numbers of requests for diagnoses and the time-consuming diagnostic process leads to longer waiting times for individuals to be assessed for ASD. However, earlier diagnosis is highly desirable as it allows for earlier allocation of specific care. It also helps the patients, who are not suffering from ASD to allow to explore possible other treatable diseases. Taken together, the development of a potent screening instrument remains an important research goal.

To address the current disparity between the number of adults seeking autism assessment and the actual number of available assessment appointments, the research groups of are not suffering from ASD to allow to explore possible of Cologne and Freiburg sought to explore the relevance of the existing Ritvo Autism Asperger Diagnostic Scale—Revised (RAADS-R) based on the recommendation provided in the German S3-guidelines on ASD diagnosis over life time [31].Accordingly the aim of this project was to create and validate a German version of the RAADS-R.

The original questionnaire was proposed in English and tested in Canada, UK, USA, and Australia [1]. Estimating an information gap in the questionnaire tools for autism at that time, the RAADS-R was validated and published in 2010 by Ritvo and colleagues. In addition to information on social relatedness, circumscribed interests, and language, which is also provided by other questionnaires, the RAADS-R includes twenty questions on sensory and motor characteristics [1]. These symptoms are relevant and entered diagnostic criteria of DSM-5 in 2013 and the ICD-11 in 2022 [3]. The multicenter study by Ritvo et al. showed good results at a cut-off of 65 with a sensitivity of 97%, a specificity of 100%, and a test–retest reliability of r = 0.987. This led to the questionnaire being included in the recommendation of current NICE guidelines [2]. Unlike many other questionnaires, the RAADS-R was validated with a clinician present to guide the participant through the questionnaire [1].

Further studies applying the RAADS-R have shown a wide range of results. A British study reported a sensitivity of 95% and a specificity of 71% [32]. The validation of a Swedish version of the questionnaire showed a sensitivity of 91% and a specificity of 93% in 272 participants [33]. A Dutch version of the RAADS-R presented a sensitivity of 73% and a specificity of 58% for the cut-off at 98 [34]. The translation of the questionnaire into French showed a sensitivity of 99% and a specificity of 67.8% in the validation study with 307 test participants. In this study, the false positive rate for patients with psychiatric disorders was 55.6% [35].

In our study, we aimed to validate a newly translated German version of the RAADS-R referred to in the following as RADS-R. Our goal was to establish a useful screening tool that could help in the decision making process for a valid diagnosis and possibly speed up the diagnostic process. Therefore, we used it as a classical self-rating instrument without presence or assistance of a clinician. Even though the original name suggests the tool to be used only for Asperger’s syndrome, following latest developments in DSM-5 and ICD-11, we employed it as a potential tool in the broaden concept of ASD. We therefore propose to change the name to RADS-R (Ritvo Autism Diagnostic Scale—Revised), a change approved by the original author of the RAADS-R, Ariella Ritvo.

## Methods

### Participants

Participants were recruited from two departments, each with an outpatient clinic for persons with ASD in adulthood (Departments of Psychiatry at the University Hospitals Freiburg and Cologne). They were divided into three groups: (I) participants with a preexisting diagnosis of ASD (n = 110), (II) participants recruited outside the ASD outpatient clinics with a diagnosis of any primary mental disorder (n = 64), and (III) participants recruited outside the ASD outpatient clinics with neither ASD nor a primary mental disorder (n = 125). All groups were recruited by contact persons either via personal contact, the outpatient-clinic, our clinical wards or through a description of the study on the university hospital website. Upon agreement, the screening via IQ tests and SCID I were performed, and questionnaires were sent by mail for participants to complete. IQ tests for all participants of the Cologne sample included the Wechsler Intelligence Test (WIE) [36] and the Mehrfach Wortschatz Intelligenztest (MWT-B) [37], a test of crystalline intelligence. For the Freiburg sample, the Culture Fair Intelligence Test (CFT-20-R) and the MWT-B were used. To increase the sample size, 35 participants of group I of the Freiburg center completed the questionnaires remotely without undergoing IQ testing. There was no screening for personality disorders in any of the group, though most participants in group I were diagnosed at one of the two study centers which take personality disorders into consideration as a potential differential diagnosis.

### Inclusion criteria

Inclusion criteria were defined as follows: All participants had to be between 18 and 65 years old and German native speakers. Participants of group III were screened for psychiatric disorders with the Structured Clinical Interview for DSM-5 (SCID I) [38]. Any participants identified with a psychiatric disorder were moved to group II.

### Instruments

The original RAADS-R was initially translated by the research group into German. This version was translated back into English by a bilingual native speaker. The re-translated version was compared with the original and approved by Riva Ariella Ritvo, main author of the RAADS-R. The final version included additional demographic questions as well as questions about previous psychiatric and neurological records of the participants and their children. The main part of the questionnaire consisted of 80 questions with four response options:

- True now and when I was young
- True only now
- True only when I was younger than 16
- Never true

The questions themselves were primarily grouped into four categories by the original authors: social relatedness (39 questions), circumscribed interests (14 questions), language (7 questions), and sensory motor (20 questions) [1].

In addition to the screenings used for the inclusion criteria and the RADS-R questionnaire, participants completed the following questionnaires: AQ, EQ [39, 40], and the Social Responsiveness Scale (SRS) Version 1 which was to be filled out by a person with close contact to the participant in the past six months [41]. Some participants with ASD had difficulty finding someone for this purpose, so their participation was accepted even when the SRS could not be provided.

### Ethical considerations

The study was carried out in accordance with the Code of Ethics of the Declaration of Helsinki and was approved by the Ethics Committees of the Medical Faculties of the University of Freiburg (503/15) and the University of Cologne (16/089). The study was registered in the German Clinical Trials Register (DRKS00010622). All participants provided written consent to participate in the study.

### Data analysis

R Version 4.1.0 and R Studio Version 1.4.1717 were used for the statistical analyses of the data. Additional packages used included caret (Version 6.0–88 [42];), pROC (Version 1.18.0 [43];), psych (Version 2.1.6 [44];), tidyverse (Version 1.3.1; [45]), and lavaan (Version 0.6–18 [46];). At collection, the data of the two centers were anonymized and merged into a single dataset. The data preprocessing included the recoding of the 17 inverse items and the calculation of the total RADS-R score and the four subscales’ total values. Participants with more than three missing items were excluded from the calculation of the total sum score. To compare the mean values of the RADS-R score between the three groups a one-way analysis of variance (ANOVA) was applied. In addition, the adjusted means of intelligence quotient and age were compared using an analysis of covariance (ANCOVA). To assess the predictive power of the RADS-R scale, a receiver operating characteristic (ROC) curve was plotted, and an optimal threshold was identified using the closest-topleft method. Applying this procedure, sensitivity and specificity were considered equally important. In addition, sensitivity, specificity and accuracy of the RADS-R score were calculated and compared to the values of the other instruments, AQ, EQ and SRS, in all participants. Lastly, both exploratory and confirmatory factor analyses were conducted. To test the theoretical four-factor structure of the scale, we conducted a confirmatory factor analysis (CFA) using maximum likelihood estimation with listwise deletion to handle missing data. Additionally, an exploratory factor analysis (EFA) was carried out using oblique rotation under maximum likelihood estimation. A scree plot of eigenvalues was used to determine the number of factors for the analysis. All tests were conducted using a two-tailed alpha level of α = 0.05.

## Results

### Demographics

Table 1 contains descriptive information about the study sample including sample size, sex, age, and IQ clustered by the three groups. Analyses show that all three groups neither differ in a statistically significant way in age (*F*_(1, 296)_ = 0.098, *p* = 0.755) nor in the sex distribution (*χ*^*2*^ = 5.80, *p* = 0.055). In IQ, however, there are significant differences between the three groups (*F*_(2, 253)_ = 13.72, *p* < 0.001). A Tukey post hoc test shows that the PMD group in particular differs from the other two groups with a significantly lower IQ.

**Table 1:** Demographic characteristics of the study sample

Group	N	Males		Females			Age		IQ	
		N	%	N		%	Mean	SD	Mean	SD
ASD	110	63	57.27	47		42.73	40.10	13.01	113.63	16.53
PMD	64	25	39.06	39		60.94	36.64	13.57	101.00	12.78
Control	125	68	54.40	57		45.60	36.86	13.57	112.43	15.12
Full sample	299	156	52.17		143	47.83	37.99	13.42	110.29	15.82

Demographic characteristics of the sample included in RADS-R comparison										
Group	N	Males		Females		Age		IQ		
		N	%	N	%	Mean	SD	N	Mean	SD
ASD	106	62	58.49	44	41.51	39.76	12.98	73	114.07	16.60
PMD	63	24	38.10	39	61.90	36.27	13.35	55	101.11	12.87
Control	124	67	54.03	57	45.97	36.70	13.51	123	112.54	15.14
Full sample	293	153	52.22	140	47.78	37.72	13.33	251	110.48	15.88

### Group Comparisons of RADS-R Scores

The mean total RADS-R scores per group and the results of the one-way ANOVA comparison across the three groups are displayed in Table 2. All three groups differ significantly from each other in the total RADS-R score (*F*_(2, 135.47)_ = 395.96, *p* < 0.001). The age and IQ adjusted ANCOVA still indicates significant differences between the groups. While IQ was not a significant covariate (*p* = 0.698), age showed a significant relation with the RADS-R total score (*p* < 0.001). Therefore, the correlation between age and RADS-G score was calculated for all three groups separately. Only in the ASD group an increase in age was positively correlated with an increase in mean RADS-R scores (Pearson's correlation *r* = 0.32, *p* < 0.001).

**Table 2:** One way ANOVA comparison of RADS-R total score and statistical characteristics of participant groups

Group	N	Mean	SD	95% CI		F	df1, df2	p
				LL	UL			
ASD	106	146.26	41.38	138.29	154.23	395.96	2.00, 135.47	< .001
PMD	63	50.37	29.88	42.84	57.89			
Control	124	23.79	18.62	20.48	27.10			

Six persons were excluded for the calculation of the RADS-R total score due to too many missing values. Therefore, the *N* in this table differs from the total *N* (see Table 1)

At both sites the described significant differences between groups could be observed, whereas these differences varied between the two centers. This can be attributed to a lower mean RADS-R score in the primary mental disorders group from Cologne compared to Freiburg. The mean RADS-R total scores by the research center and by group are shown in Table 3.

**Table 3:** RADS-R total score by research center and by group

Research center	Group	N	RAADS-R scores			95% CI	
			Mean		SD	LL	UL
University Hospital Cologne (*n* = 78)	ASD	26	155.58		29.40	143.70	167.45
	PMD	24	42.79		24.07	32.63	52.95
	Control	28	30.96		21.97	22.45	39.48
University Hospital Freiburg (*n* = 221)	ASD	84	143.24	44.28		133.37	153.10
	PMD	40	55.03	32.36		44.53	65.52
	Control	97	21.70	17.09		18.24	25.16

*CI* confidence interval, *LL* lower limit, *UL* upper limit

Significant differences between the groups were also observed in the sum scores of the four dimensions (Sensory Motor: *F*_(2, 148.04)_ = 208.9, *p* < 0.001; Language: *F*_(2, 144.32)_ = 141.81, *p* < 0.001; Social Relatedness: *F*_(2, 126.43)_ = 410.58, *p* < 0.001; Circumscribed Interests: *F*_(2, 137.63)_ = 211.51, *p* < 0.001). Again, posthoc tests showed significant differences between all three groups in all subscales’ mean scores. Table 4 contains the mean RADS-R sum scores per subscale and group.

**Table 4:** Statistical analysis of the sum scores of the subscales per group

Group	Subscale	Mean	SD	95% CI	
				LL	UL
Autism spectrum disorder (total *n* = 110)	Social (*n* = 104)	74.90	21.72	70.68	79.13
	Sensory motor (*n* = 106)	32.89	11.77	30.62	35.15
	Circumscribed interests (*n* = 106)	27.68	9.88	25.78	29.58
	Language (*n* = 107)	11.12	5.07	10.17	12.11
Primary mental disorders (total *n* = 64)	Social (*n* = 62)	23.87	18.20	19.25	28.49
	Sensory motor (*n* = 63)	12.08	8.28	9.99	14.16
	Circumscribed interests (*n* = 61)	10.31	7.47	8.40	12.22
	Language (*n* = 63)	3.76	3.10	2.98	4.54
Control group (total *n* = 125)	Social (*n* = 124)	9.31	9.38	7.64	10.97
	Sensory motor (*n* = 123)	6.54	6.52	5.38	7.71
	Circumscribed interests (*n* = 124)	5.75	5.12	4.84	6.66
	Language (*n* = 124)	2.15	2.34	1.73	2.56

A total of 12 individual comparisons were calculated, wherefore the critical p-value was adjusted according to the Bonferroni correction to *p* = .0042. Nevertheless all three groups differ significantly from each other in all four subscales except for the language score between primary mental disorders and control group with *p* = .014

### Diagnostic accuracy (Sensitivity and specificity)

**Fig. 1:**
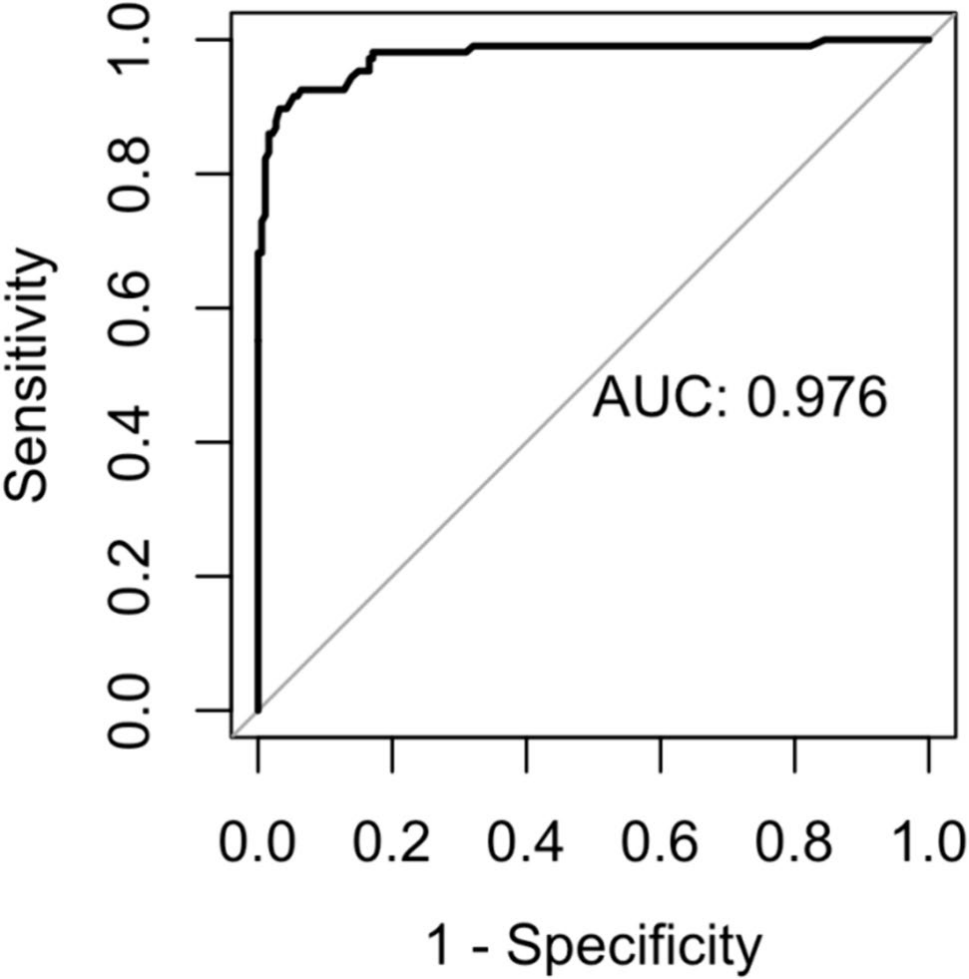
Receiver operating characteristic (ROC) curve including area under the curve (AUC). *Note*. Visual representation of the relation between sensitivity and specificity of the RAADS-R. This results in an optimal threshold of 81 with a specificity of 93.6% and a sensitivity of 92.5%

The total RADS-R scores in this sample ranged from 10 to 222 in the ASD group, 10–136 in the primary mental disorders group, and 0–93 in the control group. For further analyses of the diagnostic accuracy of the RADS-R, the data of the primary mental disorders and control groups were merged into one group of non-ASD. The ROC curve (Fig. 1) of the RADS-R total value approximates closely the upper left corner, which indicates high general sensitivity and specificity values. The area under the curve (*AUC* = 0.976) is very large and indicates a high quality and discriminatory power of RADS-R. Furthermore, the ROC curve analysis showed that the best threshold to distinguish between the ASD and non-ASD groups in the German version of the RADS-R is a score of 81. Applying this threshold to the sample of this study, a total of 92.5% of the ASD group were classified correctly as autistic (sensitivity = 0.925). In addition, 93.6% of the non-ASD participants were correctly predicted as persons without ASD (specificity = 0.936). Only 8/ 106 persons with ASD (7.5%) would have been incorrectly missed (“false negatives”), and 12/187 persons without ASD (6.4%) would have been incorrectly classified as autistic (“false positives”). Since the diagnosis of ASD was given prior to the study, the result of the RADS-R had no influence on the determination of the diagnosis.

### Comparison with other screening instruments

**Fig. 2:**
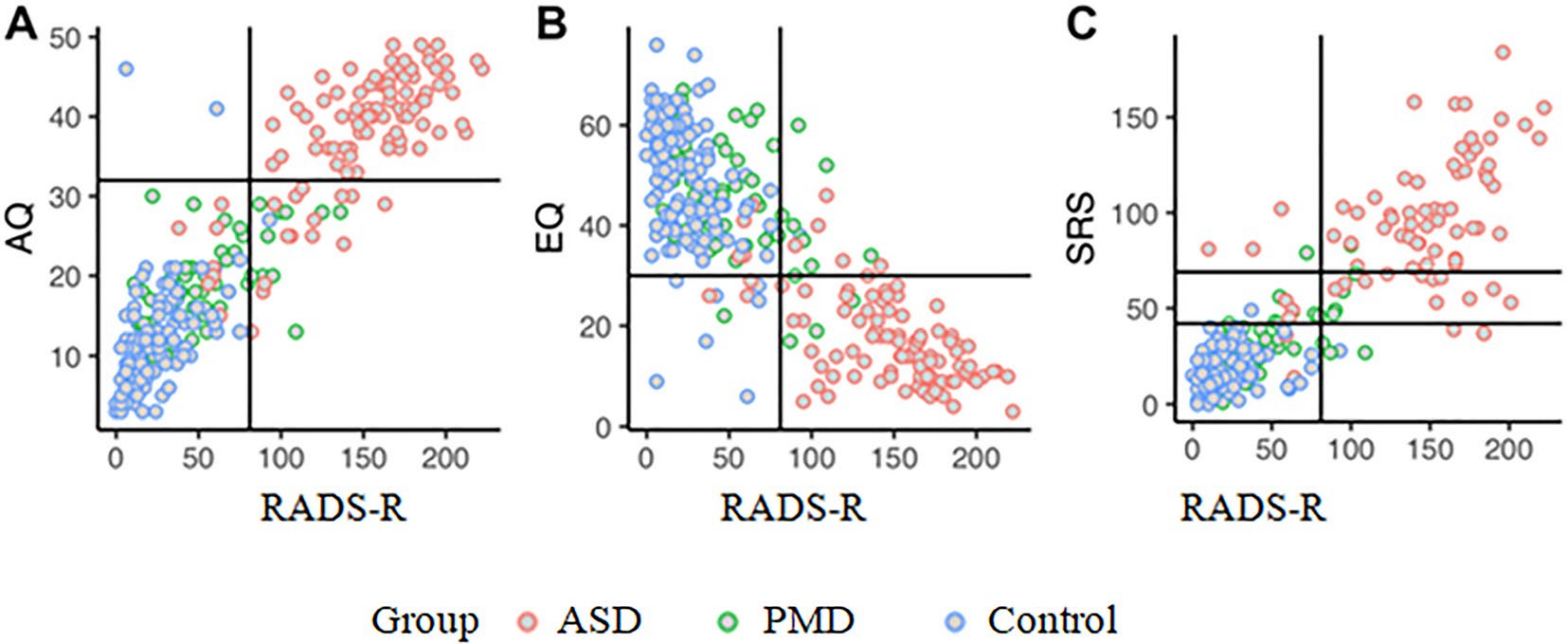
Relationship between RADS-R and AQ, EQ & SRS including thresholds. *Note*. The colors are coding for the three groups (red = ASD, green = primary mental disorders, blue = Control). Vertical and horizontal lines mark the threshold of the respective instrument. For the SRS the two most conservative values for the first and second standard deviation according to the norm tables were considered, as not only parents filled in the questionnaire and a uniform value was chosen for all. The sample size differs between the three figures because AQ, EQ and SRS data were not collected for all participants (AQ total N = 289 [104 ASD, 62 primary mental disorders, 123 Control]; EQ total N = 289 [102 ASD, 63 primary mental disorders, 124 Control]; SRS total N = 204 [77 ASD, 42 primary mental disorders, 85 Control])

Figure 2 shows the relationships between the RADS-R and the three further assessed screening instruments AQ, EQ and SRS. The RADS-R score correlates highly significantly with all three instruments (RADS-R and AQ: *r* = 0.92, *p* < 0.001; RAADS and EQ: *r* = -0.83, *p* < 0.001; RAADS and SRS: *r* = 0.85, *p* < 0.001) which indicates a high construct validity.

The sensitivity of the AQ at a threshold of 32 reaches 79.8% and the specificity 98.9% in this sample. Thus, the AQ demonstrates a better specificity but a noticeably poorer sensitivity than the RADS-R. 21 of 104 persons with ASD (20.2%) were not recognized as such by the AQ, but only 2/185 persons without ASD (1.1%) were mistakenly classified as autistic. However, the sample of people with ASD in the Cologne group had the entry requirement of having an AQ of or above 26 points to be invited to join the initial diagnostic process. This could explain the high results for specificity of the questionnaire.

The sensitivity of the EQ at a threshold of 30 reaches 90.2% and the specificity 94.1% in this sample. The EQ shows a slightly worse sensitivity and slightly better specificity than the RADS-R. 10/ 102 ASD subjects (9.8%) were not identified as such by the EQ, while 11 / 187 non-autistics (5.9%) were misclassified as autistics.

For the SRS two thresholds were considered according to the SRS norm tables. We used the most conservative thresholds for the analysis because the individuals who completed the SRS questionnaire were not only parents (see more information in Fig. 2). The sensitivity of the SRS at a threshold of 42 was 94.8% and the specificity 90.6%. The SRS thus has a better sensitivity and a worse specificity than the RADS-R. 4/77 autistic persons (5.2%) were not recognized as such by the SRS, 12/127 non-autistic persons (9.4%) were wrongly classified as autistic. Considering an SRS threshold of 69 the sensitivity reduces to 76.6% while the specificity rises to 98.4%. The SRS thus has a worse sensitivity and a better specificity than the RADS-R.

### Factor analysis

Both CFA and EFA were carried out to determine whether the assumed structure of the four subscales of the questionnaire is also reflected in the data. The model fit indices of the CFA indicated that the four-factor structured model did not fit the data adequately. The Chi-square test was significant (*χ*^2^ = 6055.63, *p* < 0.001), suggesting a significant discrepancy between the observed and model-implied covariance matrices. Both CFI and TLI were below the commonly accepted threshold of 0.90 (CFI = 0.798 and TLI = 0.792, respectively) indicating a suboptimal fit. The RMSEA was 0.059, which was just below the threshold of 0.06. The SRMR was 0.053, which is below the threshold of 0.08. All factor loadings were statistically significant (*p* < 0.05), indicating that the items were good indicators of their respective factors. However, the standardized factor loadings varied, with many loadings above 0.4, which is generally considered acceptable. The covariances between the latent variables were very high, ranging from 0.922 to 0.979, indicating substantial multicollinearity among the factors. This high degree of correlation suggests that the factors are not entirely distinct from each other. Together with the suboptimal fit of the model, the results suggest that the four factors structure might not being the most ideal structure to describe the data.

Additionally, an EFA was performed. The Kaiser–Meyer–Olkin value of *KMO* = 0.96 was quite high and therefore an exploratory factor analysis seemed to be reasonable. The Bartlett test was significant too (*χ2* = 17,420.25, *p* < 0.001) and thus confirms the assumption that the items correlated with each other. In line with Ritvo et al. an oblique rotation was carried out, as the method assumes the underlying factors to be interrelated [1]. The eigenvalues and the scree plot indicated a two-factor structure of the questionnaire. The results of the analysis showed that these two factors explain 33.9% of variance and the model does not fit the data perfectly (*χ2* = 4980.17, *p* < 0.001). Additionally, a second factor analysis was carried out with the content-wise structure of four factors appearing very reasonable from a clinical perspective. Again, the model does not fit the data perfectly (*χ2* = 4249.97, *p* < 0.001) and only explains 32.6% of variance. Therefore, the assumed structure of four factors (as in the original paper) could not be replicated in this data. Some items of the RADS-R loaded differently in the factor analysis in our statistical analysis. This is the reason for the lower explanation variance. Nevertheless, for reasons of comparability, we retain the four-factor solution of the original article [1].

### Comparison to the RAADS-14 Screen

**Fig. 3:**
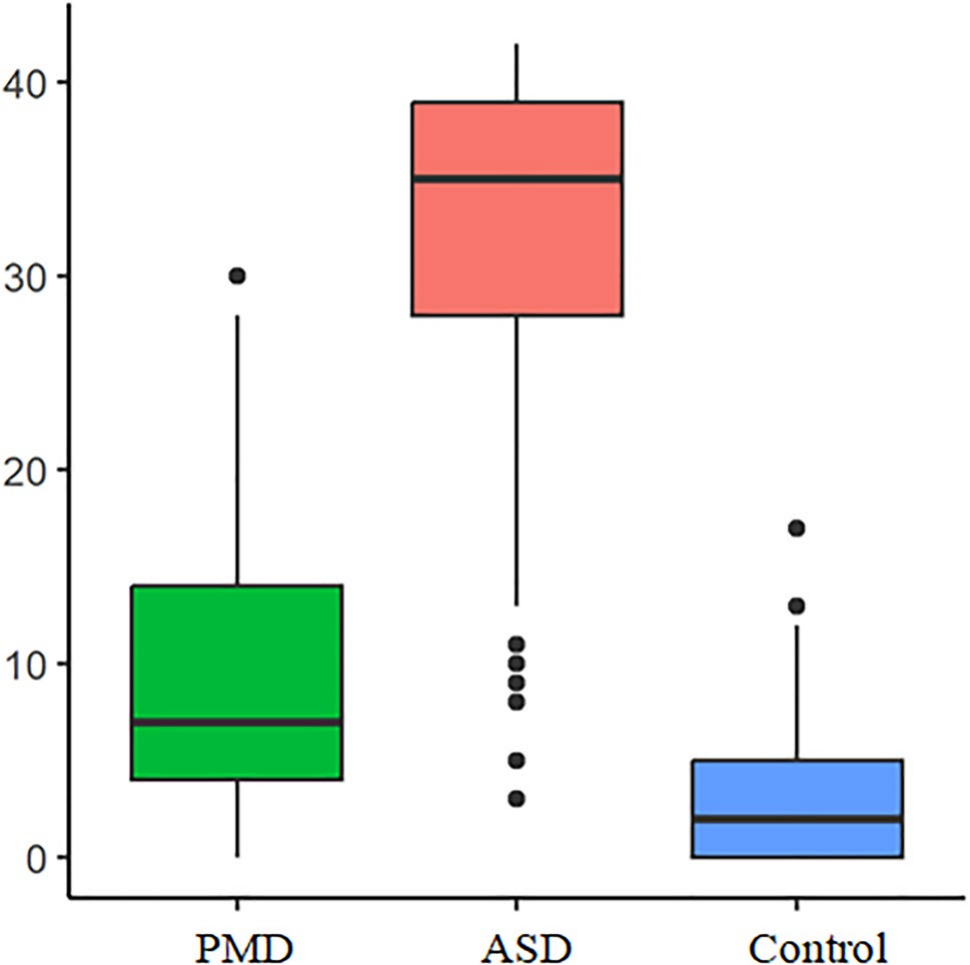
Total sum for each group in the RADS-14

Since previous research has already established a shortened form of the RAADS-R in the Swedish version, namely the RAADS-14 Screen, a comparison to the same items in the German version was carried out [47]. The RAADS-14 used the items numbered 5, 9, 25, 27, 29, 30, 42, 45, 55, 57, 58, 60, 64, 76. The following analysis showed a good distinction for the total sum between the three groups with the values significantly differing from each other in the Tukey-Test (Fig. 3).

**Fig. 4:**
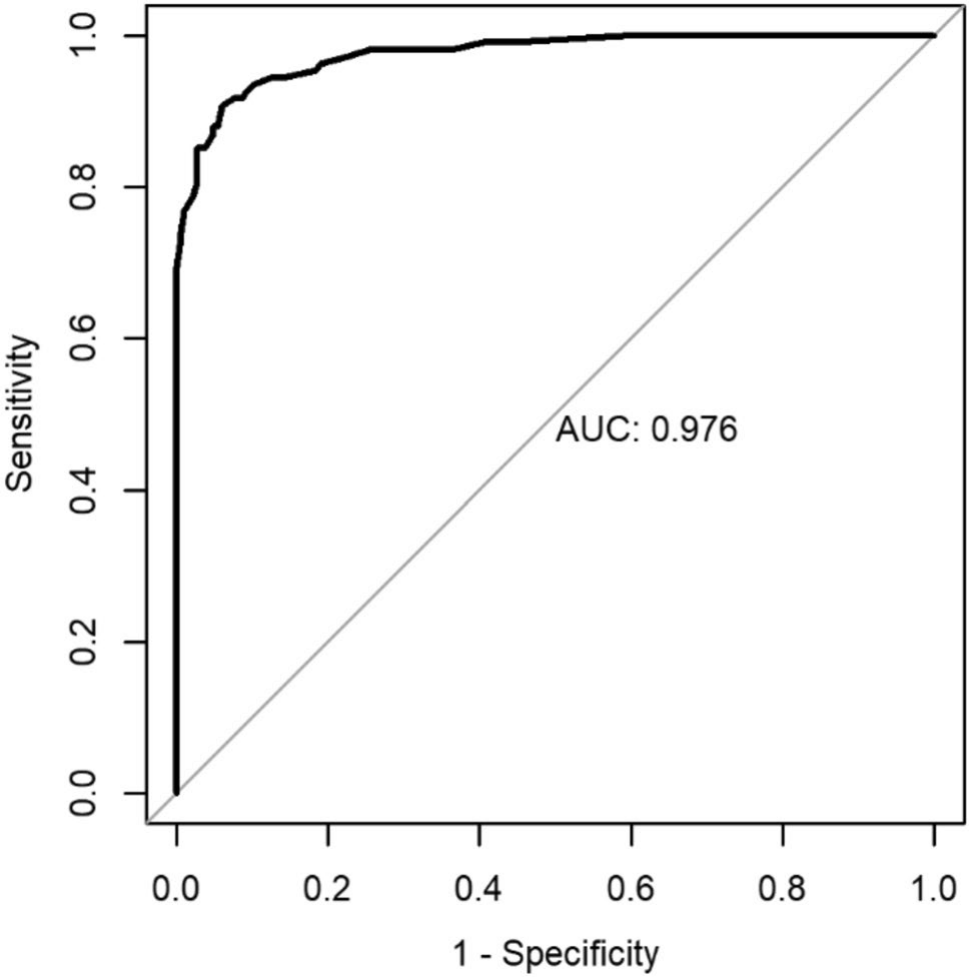
ROC-Curve for the RADS-14

The ROC-Curve for the version with 14 items proved to be satisfactory as well and showed the best cut-off at 17.5 (Fig. 4).

**Fig. 5:**
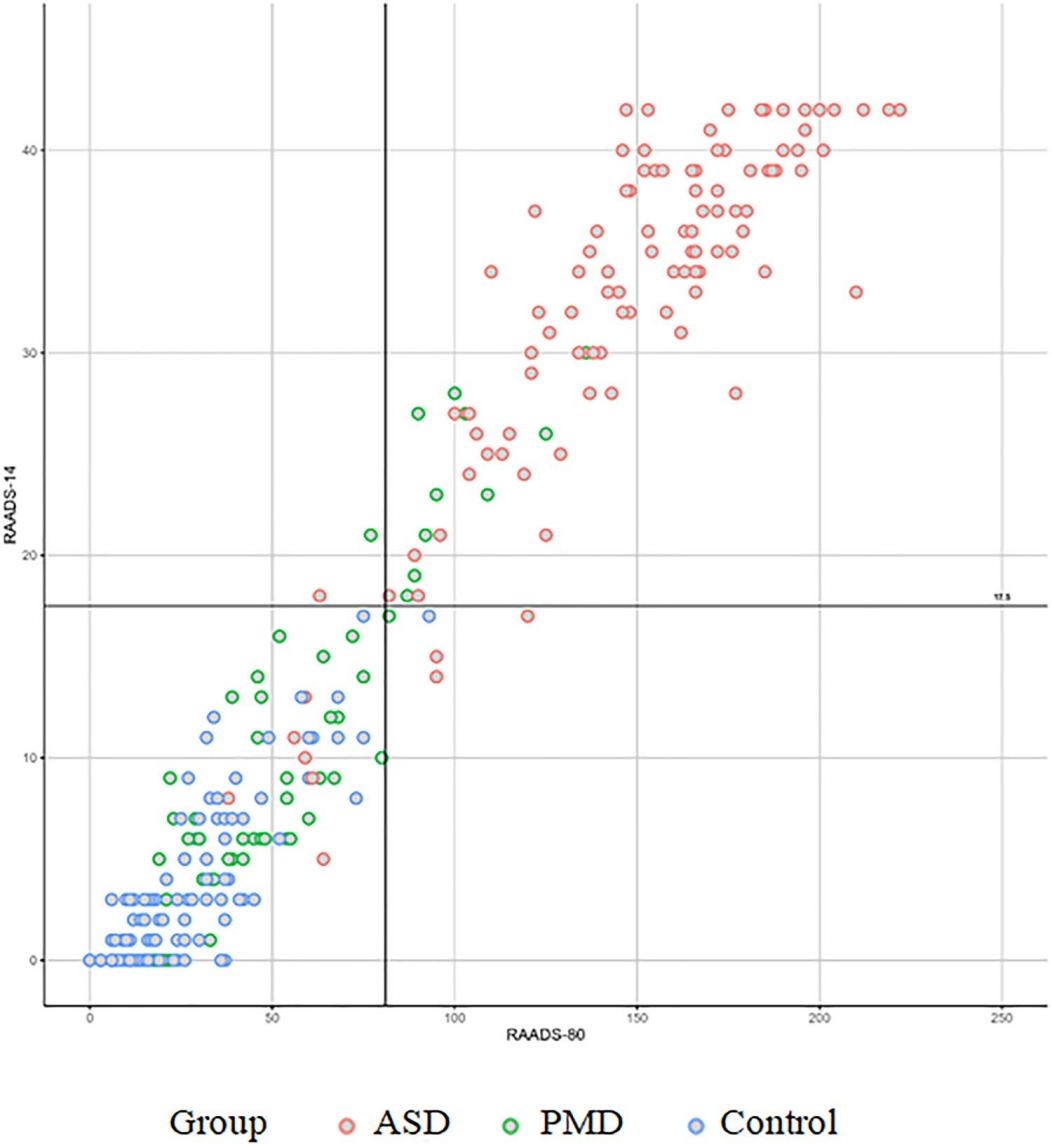
Comparison between the version with 80 items and the version with 14 items with their respective cut-offs (81, 17.5)

Due to the excellent results a comparison between the two versions of the questionnaire (80 and 14 items) was performed. This showed a very high correlation between the two versions which was highly significant (r = 0.97; p < 0.001). However the version with 14 items displayed a slightly worse sensitivity while at the same time having a higher specificity than the version with 80 items (Fig. 5).

## Discussion

The German version of the RADS-R replicated excellent result, with a high sensitivity of 92.5% and a high specificity of 93.6%, comparable to the RAADS-R. While these values are remarkably high, they do not reach the original version’s sensitivity of 97% and a specificity of 100%. Although this could theoretically be due to systematic differences in diagnostic procedures and norms between the different subsamples, this is unlikely, given that participants of the study of the English version were recruited from three different continents. The most plausible explanation for this slight difference in sensitivity and specificity is that in our German validation study, there were no clinicians present to assist in completing the questionnaire. However, the absence of a clinician was an intended since we aimed to validate the instrument as a self-rating tool. In clinical practice, it is more practical if patients fill out the questionnaire at home prior to the clinical assessment. Thus, we believe the validation of the instrument as a self-rating tool outweighs the minor reduction of sensitivity and specificity, putatively due to the absence of a clinician.

Our results are in line with the French and Swedish validation studies. Only the study by Jones and colleagues shows vastly different results with a specificity of 3,03% (13,15,17). This difference can be attributed to different sample definition. While the validation studies recruited persons with an established diagnosis of ASD or without, the study by Jones and colleagues recruited service users in the queue of undergoing ASD assessment [48]. This of course is a more realistic scenario to explore the value of the RADS-R as screening tool. At the same time, this help-seeking population might have different motivations for completing the questionnaire, possibly leading to bias. As mentioned earlier, an official diagnosis can provide certain benefits and justify support from the social and health care systems. Interestingly, in recent years, autism has become less stigmatized than other psychiatric diagnoses, such as schizophrenia or personality disorder, and has even become more attractive to many people due to popular movies, modern media and health awareness campaigns [49, 50]. The diagnosis not only opens the door to a possible support network, but also provides the person and their environment with an explanation of why certain behavioral traits exist and why certain biographical events occurred as they did. This could well lead to an aggravation bias in people expecting to receive the diagnosis. However, this bias does not only play in role in responding to questionnaires but also during clinical interviews. Furthermore, it was never the intention of the original authors of the RAADS-R to replace the clinical interview with a questionnaire, but rather to use it as a tool to assist in the diagnostic process [1].

We explored the RADS-R as a screening tool and in line with prior research found that the version with 14 items showed promising results, significantly correlating with the full 80-items version and demonstrating an even higher specificity [47]. Considering the number of questionnaires and paperwork patients have to fill out and the potential overload that come with it, it may be worth considering using the shortened version in further clinical research.

An unexpected finding of our study was the correlation between age and higher RADS-R scores. This warrants further considerations. One possible explanation could be that the older participants had more time in their life to think about their characteristic and possivly deviating personality properties. Another explanationrefers to our recruitment procedure. The contact for study participation was established either by persons who were still in treatment in the two different departments in Cologne and Freiburgor others who visited our website looking for new studies. Thereby the recruitment mainly took place amongst people who were still attached to the clinical departments. While less severely affected patients were more likely to lose contact to the diagnosing institution, those more severely impaired often stay in touch more often because they need substantially more support. Therefore, our population sample of older people with ASD could in fact have more severe symptoms than our younger group.

## Limitation

In this study, the German version of the RADS-R questionnaire was validated in three groups: people diagnosed with ASD, people diagnosed with a primary mental disorders, and people without any mental disorder. These groups obviously do not cover the heterogeneity of psychiatric diagnoses. As realized in ICD-11, there are boundaries not only to normality (broader autism phenotype) but also to many other psychiatric conditions not represented in our sample, e.g. different personality disorders and other developmental disorders (most notably ADHD). Identifying these comorbidities and differential diagnoses is part of the clinical diagnostic work-up. Questionnaires like the RADS-R are not developed for this purpose.

Additionally, a self-report questionnaire, in the way the RADS-R was used in our study, does bear the possibility of false representation of the symptoms. Underreporting could result from participants who do not understand or are unable to relate the questions to their own life (e.g., thinking in a too concrete manner and failing to transfer the question to their real life setting). Overreporting could result from a person aggravating existing peculiarities. This would most likely be the case with people with a preference for a diagnosis of ASD. As the study was carried out amongst volunteers and group II and III did not register for a diagnostic assessment, this effect is unlikely to have played a significant role in our study setting.

## Conclusion

In this study, we were able to validate the German version of the RADS-R. It achieved a very high sensitivity of 92.5% and a high specificity of 93.6% using the RADS-R as a screening instrument filled out by the patient alone with no assistance by any other person (e.g. clinician). The data suggest that RADS-R is a feasible screening instrument for ASD in the German language. In contrast to most other competing instruments, it also includes questions in the area of sensorimotor symptoms as well as information about the longitudinal presence of symptoms. As such it has a high potential for clinical practice. Projects for future research should focus on the a validation of the RADS-R of a diagnosis-seeking population in a prospective manner.

## Data Availability

The datasets used and/or analysed during the current study are available from the corresponding author on reasonable request.

## Abbreviations

3Di-Adult: Developmental, dimensional and diagnostic interview—adult version
ADI-R: Autism diagnostic interview revised
ADOS: Autism diagnostic observation schedule
ASAS: Australian scale for asperger’s syndrome
ASD: Autism spectrum disorder
AQ: Autism spectrum quotient
CATQ: Camouflaging autistic traits questionnaire
CFT-20-R: Culture fair intelligence test scale 2
EPAS: Expectations psychotherapy autism spectrum
EQ: Empathy quotient
EVA&SCOTT: “Social cognition training tool”, and its enhancement “Emotionen verstehen und ausdruecken” understanding and expressing emotions
FASTER: Freiburger asperger-spezifische therapie für ERwachsene
FQLP: Freiburg questionnaire of linguistic pragmatics
GQ-ASC: Girls questionnaire for autism spectrum condition
MWT-B: Mehrfach wortschatz test
PMD: Primary mental disorders
RAADS-R: The ritvo autism asperger diagnostic scale-revised
RADS-R: Ritvo autism diagnostic scale-revised
SAPAS: Standardised assessment of personality—abbreviated scale
SCL-K9: Symptom-Checklist-K-9
SRS: Social responsiveness scale
WIE: Wechsler intelligence test
